# Double versus triple arthrodesis for adult-acquired flatfoot deformity due to stage III posterior tibial tendon insufficiency: a prospective comparative study of two cohorts

**DOI:** 10.1007/s00264-021-05041-1

**Published:** 2021-04-25

**Authors:** Amr A. Fadle, Wael El-Adly, Ahmed Khalil Attia, Mo’men M. Mohamed, Aly Mohamadean, Ahmed E. Osman

**Affiliations:** 1 Orthopedic Department, Assiut University, Assiut, Egypt; 2 Orthopedic Surgery Department, Hamad Medical Corporation, PO Box 3050, Doha, Qatar

**Keywords:** Arthrodesis, Adult-acquired flatfoot deformity, Stage III posterior tibial tendon insufficiency, Progressive collapsing foot deformity

## Abstract

**Purpose:**

The study aims to prospectively compare double and triple arthrodesis in terms of functional outcomes and deformity correction. To the best of our knowledge, this is the first prospective comparative study in the literature to date.

**Methods:**

This is a prospective comparative cohort study carried out between May 2017 and May 2019. The study was approved by the IRB at Assiut University and done according to the Helsinki declaration. Patients with AAFD stage III aged between 15 and 40 years old were assigned to double arthrodesis or triple arthrodesis. The groups were prospectively followed for one year. Primary outcomes were union rates, AOFAS scores, and radiological parameters of deformity correction on AP and lateral plain radiographs. Secondary outcomes were operative time, time to union, and complications. The double arthrodesis was done through the medial approach, while the triple arthrodesis was done through dual medial and lateral approaches. The post-operative protocol was standardized for both groups.

**Results:**

A total of twenty-three patients matched the inclusion criteria and provided their consent to participate in the study. Thirteen (all males) patients underwent double arthrodesis, while ten (nine males and one female) patients underwent triple arthrodesis. The mean age for double and triple arthrodesis was 20.15 ± 5.63 and 25.10 ± 8.36 years, respectively, and the mean follow-up lengths were 12.46 and 12.9 months, respectively. There were no statistically significant differences between both groups in age, gender, laterality, or duration of follow-up. There were no statistically significant differences between both groups in AOFAS hindfoot scores or radiographic parameters. All patients were available for the final follow-up evaluation. All patients in both groups achieved union by four months post-operatively. The mean time to union in the double and triple arthrodesis groups was 3.39 ± 0.65 vs. 3.31 ± 0.6 months, respectively, with no statistically significant differences (*p* = 0.77). The mean operative time was significantly shorter in the double arthrodesis group than the triple arthrodesis group, 55.77 ± 15.18 vs. 91.6 ± 24.14 min (*p* < 0.001), respectively. Both double and triple arthrodesis groups had a statistically significant improvement of the mean AOFAS hindfoot score post-operatively (71.46 ± 7.77 vs. 88.38 ± 3.66, *p* < 0.001) and (66.9 ± 7.69 vs. 85 ± 5.83, *p* < 0.001), respectively. In the double arthrodesis group, the mean calcaneal pitch angle increased from 11.46° pre-operatively to 19.34° (MD = 8.45°, *p* < 0.001). The mean Meary’s angle improved from − 4.19 to 2.9° (MD = 7.32°, *p* < 0.001). Hibbs angle had a mean reduction of 6.45° post-operatively (*p* = 0.069). In the triple arthrodesis group, the mean calcaneal pitch angle improved from 10.06° pre-operatively to 17.49° post-operatively (MD = 7.12°, *p* < 0.001). The mean Meary’s angle improved from − 4.72 to 2.29° (MD = 7.09°, *p* < 0.001). The mean Hibbs angle decreased from 153.07 to 142.32° (MD = 10.54°, *p* < 0.001). The double vs. triple arthrodesis groups had no statistically significant differences in AOFAS hindfoot score improvement (16.92 vs. 19.1, *p* = 0.44), respectively. The two groups had no statistically significant differences in the magnitude of correction of all the radiographic parameters.

**Conclusion:**

Double arthrodesis is an equally reliable surgical option for AAFD stage III for achieving union, improving the functional outcomes, and deformity correction as triple arthrodesis with a significantly shorter operative time in the former. The authors recommend double arthrodesis if the calcaneocuboid joint is unaffected.

## Introduction

The adult-acquired flatfoot deformity (AAFD) is a common cause of foot pain and disability, with a prevalence of up to 3% in women over 40 years old [1]. While a broad range of foot pathologies can present with this progressive deformity, posterior tibial tendon insufficiency (PTTI) is the most important contributor to AAFD [2, 3]. AAFD is characterized by progressive loss of the medial longitudinal arch. As the arch collapses, the Achilles tendon assumes a lateral position to the hindfoot leading to the valgus component of the deformity [2, 4]. PTTI was originally classified into three stages, according to the progression, by Johnson and Strom [5]. It was later modified by Myerson to include a fourth stage [6]. Stage III is defined by a rigid deformity that is not passively correctable (Table 1). At this stage, the management options are limited to arthrodesis [2]. However, a more comprehensive classification system has been recently published by Myerson et al. They also recommended renaming AAFD to progressive collapsing foot deformity (PCFD) [7].

**Table 1:** Stages of adult-acquired flatfoot deformity (AAFD)*

Stage	Deformity	Physical findings	Radiographic findings
I	PTT tendinosis/tenosynovitis No deformity	Able to do single heel raise	Xray: normal MRI: + / − PTT early degeneration
IIa	Flexible flatfoot deformity Flexible hindfoot valgus Normal forefoot	Unable to do single heel raise Mild sinus tarsi pain	Arch collapse < 30% TN uncoverage
IIb	Flexible flatfoot deformity Flexible hindfoot valgus Flexible forefoot abduction	Unable to do single heel raise Mild sinus tarsi pain Too many toes sign	Arch collapse > 30% talonavicular uncoverage
III	Rigid flatfoot deformity Rigid hindfoot valgus Rigid forefoot abduction	Unable to do single heel raise Severe sinus tarsi pain Too many toes sign	Arch collapse > 30% talonavicular uncoverage Subtalar arthritis
IV	Rigid forefoot abduction Rigid hindfoot valgus Deltoid ligament insufficiency Flexible or rigid ankle valgus	Unable to do single heel raise Severe sinus tarsi pain Too many toes sign Ankle pain and instability	Arch collapse > 30% TN uncoverage Subtalar arthritis Lateral talar tilt Ankle arthritis

Abbreviations: *PTT* posterior tibialis tendon

^*^Adapted from Johnson and Strom [5], and Myerson [6]

In modern literature, double arthrodesis has been proven to restore function and provide a plantigrade foot [16–22]. Moreover, double arthrodesis has been shown to be protective against post-operative ankle valgus when compared to triple arthrodesis [23]. The medial double technique has the advantage of a single medial incision shown to have a decreased risk of wound complications compared to the standard dual-incision triple arthrodesis [2, 19]. However, the literature remains limited in comparative studies [16, 24].

## Aim

The study aims to prospectively compare double and triple arthrodesis in terms of functional outcomes and deformity correction. We hypothesize that double arthrodesis is equivalent to triple arthrodesis in improving functional and radiographic outcomes with a shorter operative time in the former and fewer wound complications. To the best of our knowledge, this is the first prospective comparative study in the literature to date.

## Methods

### Study design

This prospective comparative cohort study was performed at a level-I academic centre with an established foot and ankle unit. The study was approved by the Institutional Review Board at Assiut University and carried out according to the Helsinki declaration. All patients who presented to the foot and ankle unit at Assiut University medical centre with PTTI in the period from May 2017 to May 2019 were evaluated clinically and radiographically. Patients with PTTI stage III were screened against the inclusion and exclusion criteria. Twenty-three patients with PTTI stage III were eligible for inclusion, all of whom provided informed consent to participate in the study. Patients were assigned to either the double arthrodesis group or the triple arthrodesis group based on the presence or absence of calcaneocuboid degenerative changes on radiographs and/or lateral joint line tenderness.

### Inclusion/exclusion criteria

Inclusion criteria were symptomatic PTTI stage III with subtalar joint arthritis refractory to prolonged non-operative management in patients aged between 15 and 45 years old. Asymptomatic patients, post-traumatic and paralytic AAFD, and PTTI stages other than stage III in patients younger than 15 or older than 45 years old were excluded.

### Outcomes

#### Clinical evaluation

All patients were examined clinically pre-operatively, immediately postoperative, and at regular intervals. To assess functional outcomes, the American Orthopedic Foot and Ankle (AOFAS) hindfoot scores were collected at the pre-operative and the final 12-month follow-up visits. The AOFAS hindfoot score is the sum of points out of a potential 100 points that assess function (50 points), pain (40 points), and alignment (10 points) [25]. Operative time was recorded intra-operatively.

#### Radiographic evaluation

All patients had weight-bearing anteroposterior (AP) and lateral foot plain radiographs pre-operatively, immediately post-operatively, and at every subsequent clinical evaluation appointment for union and alignment measures. Measurements obtained at each visit were the talus-first metatarsal angle (Simmons angle), and talonavicular coverage angle on AP views, and calcaneus-first metatarsal angle (Hibb’s angle), calcaneal pitch angle, lateral talus-first metatarsal angle (Meary’s angle), and cuneiform-fifth metatarsal height on lateral views.

### Statistical analysis

Data were analyzed using SPSS 25 (IBM, Armonk, NY). Continuous data were expressed as mean ± standard deviation (SD), while nominal data were expressed as frequency (percentage). Chi-squared (*χ*^2^) test was used to compare the nominal data of different groups in the study, while Student’s *t*-test was used to compare the mean of different groups. A two-tailed paired *t*-test was used to compare continuous pre-operative and post-operative data of the same group. *p* values were considered significant if < 0.05.

### Surgical technique

For both techniques, all patients were operated on in supine position with the application of a tourniquet. Gastrocnemius recession or percutaneous Achilles tendon lengthening was performed when needed depending on whether there was isolated gastrocnemius or Achilles tendon tightness, respectively. Double arthrodesis was done through the medial approach described by Anand et al. and Hyer et al. [17, 26]. Dual medial and lateral approaches were used for triple arthrodesis, as described by Seybold and Coetzee [27]. The subtalar joint was fixed in a 4° valgus. Achievement of a plantigrade well-aligned foot was judged intra-operatively with simulated weight-bearing using a rigid metal tray sterile cover intra-operatively. Iliac crest bone autografts were used to fill defects as needed. No allografts or bone marrow aspirate were used.

#### Double arthrodesis

After prepping and draping, a single medial approach was done along the tibialis posterior (TP) tendon, extending from posterior to the medial malleolus to just distal to the plantar aspect of the navicular tuberosity. The TP tendon sheath was opened, and the tendon was inspected and retracted posteriorly. A transverse incision of the talonavicular joint (TNJ) capsule was done followed by around the head of the talus and extended proximally to the anterior and middle facets of the subtalar joint. The TNJ was then distracted using laminar spreaders or pin distractors, and the articular surfaces of the joint were prepared for fusion using osteotomes down to bleeding subchondral bone surface. This, in turn, allows easier mobilization of the STJ (Fig. 12). Next, the interosseous ligament is transected, and the STJ is distracted, exposing the three facets. The STJ was then prepared for fusion as in the TNJ while preserving the tibiocalcaneal fibers of the superficial deltoid ligament. The joint surfaces were then drilled using a 2.5-mm drill bit to encourage union.

**Fig. 1:**
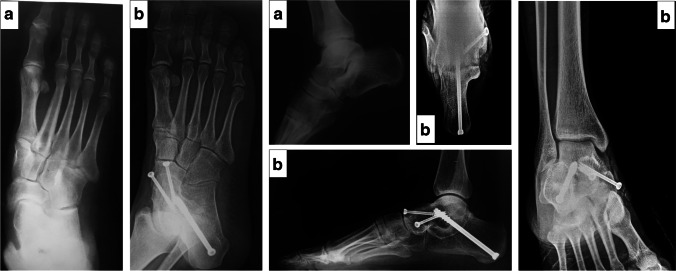
Pre-operative (**a**) and 12 months post-operative (**b**) radiographs of a double arthrodesis. This 26-year-old male patient with right-sided PTTI stage III had a significant improvement in his AOFAS hindfoot score from 60 pre-operatively to 90 post-operatively. Union was achieved at 3.5 months. No complications were reported, and the surgical time was 70 min

The TNJ and STJ were aligned to reestablish a near-normal talo-1st metatarsal angle, and guide pins were driven across these joints to maintain the reduction. The foot alignment and guide pin placement were then confirmed under fluoroscopic AP, lateral and axial views. The STJ was fixed with one or two cannulated partially threaded 7.3-mm or 6.5-mm compression screws (Synthes, West Chester, PA) from the posterior calcaneus into the talar body, with or without a screw from the anterior process of the calcaneum to the talar head. The TNJ was fixed with two or three cannulated partially threaded 4.5-mm compression screws (Synthes, West Chester, PA). If the forefoot deformity remained under-corrected or there was medial column instability, a first tarsometatarsal (TMT-1) or a naviculocuneiform (NC) arthrodesis was added accordingly (Fig. 1).

#### Triple arthrodesis

##### Lateral approach

After prepping and draping, a lateral incision is made from just inferior to the distal tip of the lateral malleolus to the base of the fourth metatarsal exposing the STJ, CC joint, and the lateral TNJ. Care is taken to avoid branches of the sural and superficial peroneal nerves running just inferior and superior to the incision. Next, deep dissection to identify the extensor digitorum brevis (EDB) muscle belly, which is followed to its origin, was carried out. The EDB origin is released, and the muscle reflected distally, allowing access to the CC joint and exposure of the sinus tarsi, and the lateral aspect of the TNJ. The Hoke tonsil is next evacuated by carefully following the contours of the calcaneus with a no. 15 blade, beginning at the anterior process. Care is taken to identify and protect the peroneal tendons. All of the sinus tarsi contents are then removed, including the interosseous ligament, to gain exposure to the anterior compartment of the STJ. A laminar spreader is placed into the sinus tarsi to distract the joint. In some cases, transection of the tight calcaneofibular (CF) ligament was necessary for adequate exposure. Articular cartilage is removed from the anterior, middle, and posterior facets with osteotomes. The calcaneocuboid joint (CCJ) is distracted and prepared in the same way, followed by the lateral aspect of the TNJ medially through the distracted CCJ.

##### Medial approach

**Fig. 2:**
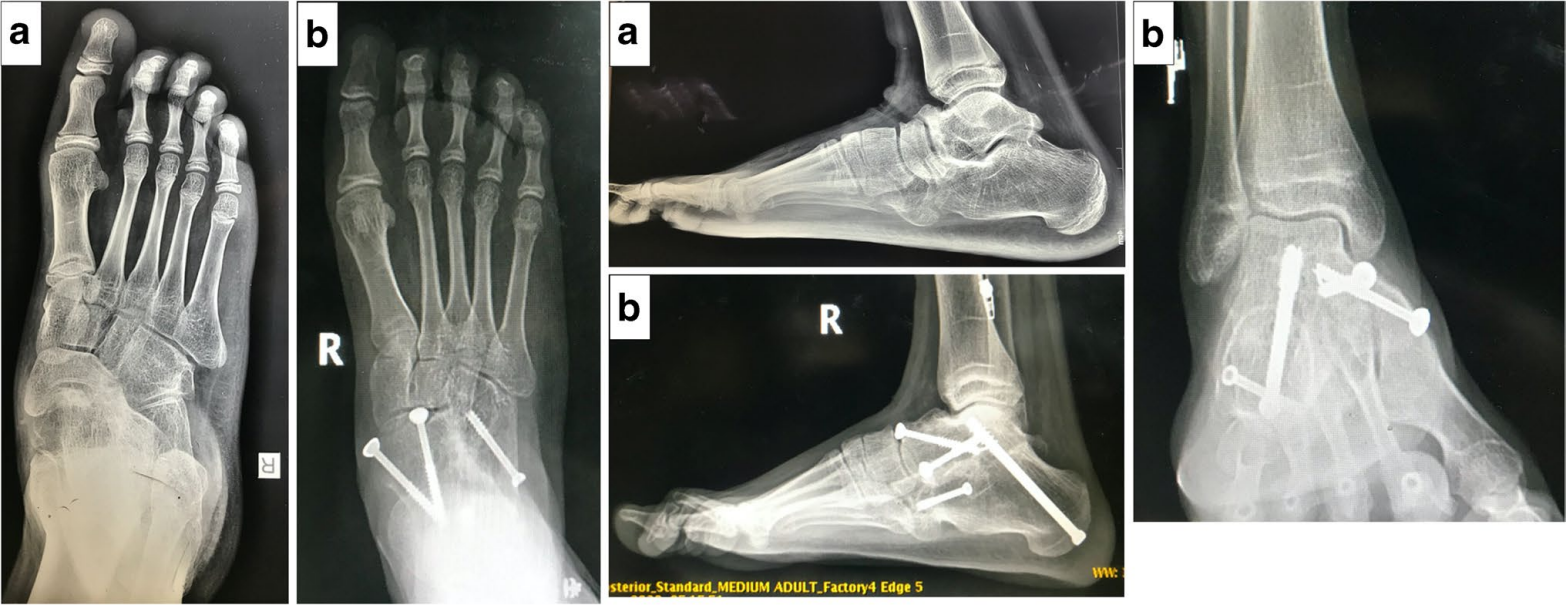
Pre-operative (**a**) and 12 months post-operative (**b**) standing foot radiographs of a triple arthrodesis. This 16-year-old male patient with right-sided PTTI stage III had a significant improvement in his AOFAS hindfoot score from 62 pre-operatively to 90 post-operatively. Union was achieved at 2.5 months. No complications were reported, and the surgical time was 120 min

A medial approach to prepare the medial TNJ and any remaining parts of the STJ is carried out as described earlier for the double arthrodesis. The joint surfaces were then drilled using a 2.5-mm drill bit to encourage union. Deformity correction and fixation were similar to the double arthrodesis with the addition of CCJ fixation using partially threaded 4.5-mm or 4.0-mm cannulated screws (Synthes, West Chester, PA) (Fig. 2).

### Post-operative care

Both groups followed the same opioid-sparing analgesia and immobilization regimen. The operated leg was placed in a well-padded below-knee backslab in a natural position for two weeks. Wound evaluation and removal of stitches were done at two weeks post-operatively, and then the slab was replaced by a below-knee cast for additional four weeks. The cast is removed at six weeks post-op for assessment and radiographs. The cast was continued for additional weeks depending on union progression. Weightbearing in regular shoes was allowed after union.

## Results

### Study population

A total of twenty-three patients matched the inclusion criteria and provided their consent to participate in the study. Thirteen (all males) patients underwent double arthrodesis, while ten (nine males and one female) underwent triple arthrodesis. The mean age for double and triple arthrodesis was 20.15 ± 5.63 and 25.10 ± 8.36 years, respectively, and the mean follow-up duration was 12.46 ± 2.88 and 12.9 ± 3.07, respectively. There were no statistically significant differences between the groups in age, gender, laterality, or follow-up duration. All patients were available for the final follow-up evaluation (Table 2).

**Table 2:** Baseline characteristics of double and triple arthrodesis groups

	Double arthrodesis (*n* = 13)	Triple arthrodesis (*n* = 10)	*p*-value
Age (Yr)^a^	20.15 (± 5.63)	25.10 (± 8.36)	0.10
Gender^b^			0.43
Male	13 (100%)	9 (90%)	
Female	0	1 (10%)	
Laterality^b^			0.19
Right	7 (53.8%)	8 (80%)	
Left	6 (46.2%)	2 (20%)	
Comorbidities	0 (0%)	0 (0%)	1.0
Follow-up duration (m)^a^	12.46 (± 2.88)	12.90 (± 3.07)	0.73

*N* number, *Yr* years, *M* months, *F/U* follow-up, *AOFAS* American Orthopedic Foot and Ankle Society, *SD* standard deviation

^**a**^Data reported as mean (SD)

^**b**^Data reported as number (percentage)

*p* < 0.05 was considered statistically significant

### Union and complications

All patients (100%) in both groups achieved union of the arthrodesis sites by four months post-operatively. The mean time to union in the double and triple arthrodesis groups was 3.39 ± 0.65 months vs. 3.31 ± 0.60, respectively with no statistically significant differences (*p* = 0.77). One patient in each group had residual pain that developed after prolonged standing and heavy manual work. No other complications were reported in the double arthrodesis group, while one 25-year-old male patient in the triple arthrodesis group had wound dehiscence of the lateral wound. He was managed by oral antibiotics and local wound care. He underwent hardware removal two years post-operatively. None of the patients in the double arthrodesis group developed CCJ tenderness or degenerative changes on the final radiographs. The mean operative time was significantly shorter in the double arthrodesis group than the triple arthrodesis group, 55.77 ± 15.18 vs. 91.6 ± 24.14 min (*p* < 0.001), respectively (Table 3).

**Table 3:** Comparison of operative time and clinical outcomes of both groups

	Double arthrodesis (*n* = 13)	Triple arthrodesis (*n* = 10)	*p*-value
Operative time (min)^a^	55.77 (± 15.18)	91.60 (± 24.14)	< 0.001
Union rate^b^	13 (100%)	10 (100%)	1.00
Union time (mon)^a^	3.39 (± 0.65)	3.31 (± 0.60)	0.77
Complications^b^	0 (0%)	1 (10%)	0.43
Residual pain^b^	1 (7.7%)	1 (10%)	0.85

*Min* minute, *mon* months, *n* number

^**a**^ reported as mean (SD)

^**b**^Data reported as number (percentage)

*p* < 0.05 was considered statistically significant

### Functional and radiographic outcomes

There were no statistically significant differences between both groups in AOFAS hindfoot scores, AP, and lateral radiographic parameters. Both double and triple arthrodesis groups had a statistically significant improvement of the mean AOFAS hindfoot total score post-operatively (71.46 ± 7.77 vs. 88.38 ± 3.66, *p* < 0.001) and (66.9 ± 7.69 vs. 85 ± 5.83, *p* < 0.001), respectively. In both groups, there were no statistically significant differences in AP talo-1st metatarsal angles between pre and post-operative radiographs within the group.

In the double arthrodesis group, the mean calcaneal pitch angle increased from 11.46° preoperatively to 19.34° post-operatively with a statistically significant difference (MD = 8.45°, *p* < 0.001). The mean talonavicular coverage angle improved from 24.08° pre-operatively to 4.36° post-operatively, with a statistically significant difference (MD = 19.72°, *p* < 0.001). The mean Meary’s angle increased from − 4.19° pre-operatively to 2.9° post-operatively, with a statistically significant difference (MD = 7.32°, *p* < 0.001). The mean medial cuneiform-5th metatarsal height increased from − 0.28 cm pre-operatively to 2.17 cm post-operatively, with statistically significant difference (MD = 2.45 cm, *p* < 0.001). Hibbs angle had a mean reduction of 6.45° ± 10.50° post-operatively, but this reduction did not reach statistical significance (*p* = 0.069) (Table 4).

**Table 4:** Pre-operative and post-operative functional and radiological outcomes of double and triple arthrodesis

	Double arthrodesis* (*n* = 13)	Triple arthrodesis* (*n* = 10)	*p*-value^a^
AOFAS Hindfoot Score			
Pre-operative	71.46 (± 7.77)	66.9 (± 7.69)	0.18
Post-operative	88.38 (± 3.66)	86.0 (± 5.83)	0.28
*p*-value^b^	< 0.001	< 0.001	
AP Talo-MT1 (Simmons) angle			
Pre-operative	14.05 (± 3.44)°	11.55 (± 3.23)°	0.11
Post-operative	14.29 (± 2.29)°	11.68 (± 2.86)°	0.04
*p*-value^b^	0.51	0.60	
TN coverage angle			
Pre-operative	24.08 (± 4.32) °	23.19 (± 4.38)°	0.63
Post-operative	4.36 (± 1.83)°	3.77 (± 1.65)°	0.43
*p*-value^b^	< 0.001	< 0.001	
Calcaneo-MT1 (Hibb’s) angle			
Pre-operative	148.70 (± 10.84)°	153.07 (± 4.47)°	0.27
Post-operative	142.78 (± 3.61)°	142.32 (± 1.72)°	0.70
*p*-value^b^	0.069	< 0.001	
Calcaneal pitch angle			
Pre-operative	11.46 (± 5.08)°	10.06 (± 2.30)°	0.46
Post-operative	19.34 (± 5.34)°	17.49 (± 2.10)°	0.28
*p*-value^b^	< 0.001	< 0.001	
Lat Talo-MT1 (Meary’s) angle			
Pre-operative	-4.19 (± 0.89)°	-4.72 (± 1.16)°	0.26
Post-operative	2.90 (± 1.09)°	2.29 (± 1.41)°	0.29
*p*-value^b^	< 0.001	< 0.001	
Cuneiform-MT5 height (cm)			
Pre-operative	− 0.28 (± 1.13)	− 0.19 (± 1.45)	0.88
Post-operative	2.17 (± 0.34)	2.23 (± 0.44)	0.80
*p*-value^b^	< 0.001	0.001	

*AOFAS* American Orthopedic Foot and Ankle Society, *MT1* first metatarsal, *TN* talonavicular, *MT5* fifth metatarsal, *AP* anteroposterior, *Lat* lateral

^*^Data reported as mean (SD)

^a^Double vs. triple arthrodesis

^b^Preoperative vs. postoperative

*p* < 0.05 was considered statistically significant

In the triple arthrodesis group, the mean talonavicular coverage angle improved from 23.19° pre-operatively to 3.77° post-operatively, with a statistically significant difference (MD = 19.41°, *p* < 0.001). The mean calcaneal pitch angle increased from 10.06° pre-operatively to 17.49° post-operatively with a statistically significant difference (MD = 7.12°, *p* < 0.001). The mean Meary’s angle increased from − 4.72° pre-operatively to 2.29° post-operatively, with a statistically significant difference (MD = 7.09°, *p* < 0.001). The mean medial cuneiform-5th metatarsal height increased from − 0.19 cm pre-operatively to 2.23 cm post-operatively, with statistically significant difference (MD = 2.38 cm, *p* = 0.001). The mean Hibbs angle decreased from 153.07° pre-operatively to 142.32° post-operatively. Contrary to the double arthrodesis group, this reduction was statistically significant (MD = 10.54°, *p* < 0.001) (Table 4).

**Fig. 3:**
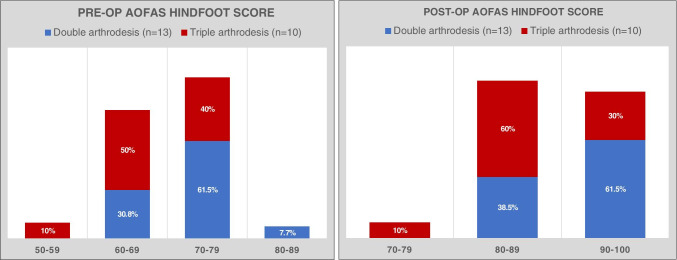
Breakdown of pre and post-operative AOFAS hindfoot score in double and triple arthrodesis groups. Abbreviations: Post-op, post-operative; pre-op, pre-operative; AOFAS, American Orthopedic Foot and Ankle Society; *n*, number

The double and triple arthrodesis had no statistically significant differences in total AOFAS hindfoot score improvement post-operatively (16.92 vs. 19.1, *p* = 0.44), respectively. Figure 3 shows the percentages of AOFAS hindfoot score distribution in each group. The two groups had no statistically significant differences in the magnitude of correction of all the radiographic AP and lateral parameters (Table 5).

**Table 5:** Comparison of the mean difference between post-op and pre-op in both groups

	Double arthrodesis^a^ (*n* = 13)	Triple arthrodesis^a^ (*n* = 10)	*p*-value
AOFAS Hindfoot Score	16.92 (± 7.12)	19.10 (± 5.63)	0.44
AP Talo-MT1 (Simmons) angle	0.48 (± 2.45)°	0.30 (± 1.55)°	0.43
TN coverage angle	19.72 (± 5.27)°	19.41 (± 3.61)°	0.87
Calcaneo-MT1 (Hibb’s) angle	6.45 (± 10.50)°	10.54 (± 3.92)°	0.31
Calcaneal pitch angle	8.54 (± 6.20)°	7.12 (± 2.44)°	0.55
Lat Talo-MT1 (Meary’s) angle	7.32 (± 1.70)°	7.09 (± 2.10)°	0.79
Cuneiform-MT5 height (cm)	2.45 (± 1.27)	2.38 (± 1.25)	0.90

*AOFAS* American Orthopedic Foot and Ankle Society, *MT1* first metatarsal, *TN* talonavicular, *MT5* fifth metatarsal, *AP* anteroposterior, *Lat* lateral

^**a**^Data reported as mean (SD)

*p* < 0.05 was considered statistically significant

## Discussion

A few studies reported on the outcomes of double arthrodesis. Most of these studies were retrospective [17–21, 24], lacked a comparative group of triple arthrodesis [17–22], and/or included a heterogeneous group of pathologies not limited to AAFD due to PTTI [18, 21]. We aimed to avoid these limitations through a rigorous methodology and inclusion/exclusion criteria.

### Functional and radiological outcomes

All patients in the double arthrodesis groups in our study achieved good to excellent AOFAS hindfoot score post-operatively with a mean of 88.4 ± 3.7 out of 94 attainable points as the six points awarded for STJ motion are lost with successful arthrodesis. This favourable outcome was consistent among most of the studies [18, 20, 21]. Notably, the AOFAS score improvement was greater in the triple arthrodesis group than the double arthrodesis group 19.1 ± 5.63 vs. 16.9 ± 7.12, respectively. However, the difference was neither clinically nor statistically significant (*p* = 0.44).

Our findings confirm that double arthrodesis has a similar magnitude of deformity correction to triple arthrodesis. Post-operatively, the double arthrodesis group had a significant correction of Meary’s angle, calcaneal pitch, medial cuneiform-5th metatarsal height, talonavicular coverage angle indicative of successful correction of the planus and abduction deformities, and restoration of the medial longitudinal arch. Our findings on deformity correction are consistent with those reported by other studies on double arthrodesis [16–18, 20–22].

### Mechanics and adjacent joint issues

A reasonable approach to correction of AAFD depends on understanding the biomechanical rationale for hindfoot joints arthrodesis. First, the talonavicular joint arthrodesis is considered the cornerstone of the deformities. In the flexible pes planovalgus deformity, TNJ arthrodesis alone was proven to be sufficient to correct all the deformities adequately [28, 29]. Moreover, fusion on the TN joint has been shown to block motion across STJ and CCJ in cadaveric studies [30, 31]. As the deformity becomes rigid, STJ arthrodesis is required for coronal deformity correction in addition to the TNJ arthrodesis [32]. This brings us to the CC joint arthrodesis. If the CCJ is not arthritic or subluxated, it should be spared. Anand et al. suggested that avoiding additional lateral column shortening caused by CC fusion in triple arthrodesis would facilitate the correction of forefoot abduction deformity [17]. Another disadvantage of the triple arthrodesis is the development of ankle and midfoot arthritis in up to 50% of cases [12, 13, 33]. Anand et al. hypothesized that the small, possibly unnoticeable, CCJ motion in double arthrodesis would help dissipate the stresses on the ankle and midfoot joints to a considerable extent, thus preventing adjacent joint arthritis [17]. This protective effect on adjacent joints is also supported by Sammarco et al. and Hyer et al. [18, 23]. Recently, Tejero et al. reported that only 4 out of 67 (5.9%) feet with double arthrodesis developed deformity progression and required ankle arthrodesis at 6.6 years of follow-up [20]. In contrast, triple arthrodesis has a reported rate of deformity progression of up to 38% [33]. In our study, the only indication for triple arthrodesis was the involvement of CCJ. Moreover, none of the patients in the double arthrodesis group developed any arthritic changes to the CCJ or lateral pain at one year post-operatively. While none of the patients in either group developed deformity progression, the duration of follow-up was not sufficient to comment on the protective effect of sparing the CCJ on the ankle and midfoot joints, which was beyond the scope of the current study.

### Surgical approach

Another potential benefit of double arthrodesis stems from using one medial incision. The foot deformity correction, especially with severe valgus, places lateral soft tissues under tension [17]. The lateral skin incision and dissection required for triple arthrodesis further compromises those soft tissues. This led to a considerable rate of lateral skin complications reported in many studies [13, 15, 34, 35]. In our study, one patient (10%) in the triple arthrodesis developed lateral wound dehiscence that resolved with local wound care. None of our patients in the double arthrodesis group developed skin complications.

### Union

We report a 100% union rate in both double and triple arthrodesis groups, and all patients retained the correction at final follow-up with no hardware failure in either group. With the exception of one study [24], all other studies reported a consistently favourable union rate in the double arthrodesis group ranging between 89 and 100% [17–20, 22]. Recently, Tejero et al. published the results of the largest cohort (67 feet) of medial arthrodesis with the most extended mean follow-up to date (6.6 years) [20]. They reported complete union in 60 out of 67 feet (89%). 4/7 nonunions developed an asymptomatic TNJ pseudoarthrosis and required no additional surgery [20]. Anand et al. reported a union rate of 16/18 (89%). One patient had isolated TNJ nonunion with minimal functional limitations and did not undergo additional surgeries. One patient developed both TNJ and STJ nonunions that were revised 16 months later [17]. Sammarco et al. reported a 15/16 (94.8%) union rate [18]. Knupps et al., Philippot et al., and Brilhault, in three different studies, reported a 32/32 (100%), 14/14 (100%), and 15/15 (100%) union rate after double arthrodesis, respectively [19, 21, 22]. This contradicts the findings of another comparative study by Burrus et al., who reported unfavourable outcomes in the double arthrodesis group [24]. Out of nine double arthrodeses, four had TN nonunion (44%), three had incomplete unions (33%), six had hardware failures (67%), and five lost the hindfoot deformity correction (56%) [24]. This difference in union rate might be attributed to the different demographics of the double arthrodesis cohorts. Advancing age has been correlated with higher rates of nonunion of hindfoot arthrodesis [36]. The mean age in their double arthrodesis group was 59.22 years, and 33% of them had rheumatoid arthritis compared to 20.15 years with no comorbidities in our cohort. Moreover, there were differences in techniques used. They used allografts while we used autografts. They used a dorsal approach to prepare the TNJ, while we used a medial approach. An anatomical study showed that the total accessible talonavicular articular surface area for the medial and dorsal approaches was 71% and 92%, respectively [37]. Another study showed that 91% of the TNJ and STJ areas could be accessed through the medial approach [38]. It is important to note that all the anatomical specimens in both studies had no deformity [37, 38]. In advanced AAFD, the hindfoot is in valgus, and the forefoot is abducted, allowing even superior access to these joints in comparison to normal feet when a medial approach is used [17].

### Efficiency

We report a significantly shorter operative time in the double arthrodesis than in the triple arthrodesis groups (55.77 ± 15.18 vs. 91.6 ± 24.14 min, *p* < 0.001). This shorter duration translates to shorter tourniquet time and possibly lower infection rate. While we did not perform a cost analysis in our institute, the double arthrodesis understandably uses fewer implants. Moreover, better operation room time utilization could possibly lead to shorter waiting lists as more procedures can be done one a given day. Galli et al. reported similar findings [39]. The mean procedure time was significantly shorter in double arthrodesis than triple arthrodesis (84 ± 29 vs. 104 ± 23 min, *p* = 0.0033). Moreover, the implants for triple arthrodesis cost, on average, 2.4 times those for double arthrodesis (*p* < 0.001) [39].

### Limitations

This study is not without limitations. First, we included a small number of patients. We intentionally set strict inclusion criteria to ensure a homogenous group of only PTTI patients excluding post-traumatic and paralytic conditions. Moreover, we only included stage III PTTI, which further limited the number of eligible patients. However, most of the published literature reported similar numbers of patients. To the best of our knowledge, our study is the first prospective controlled study to report both functional and radiological outcomes. Moreover, all of the included patients in our study were available for the final follow-up. Finally, the assessors were blinded to patient allocation on pre-operative assessment, limiting the risk of bias. Another limitation is the relatively short follow-up length. Consequently, we are unable to make conclusions about the long-term outcomes of double arthrodesis. However, most of the published literature had a similar follow-up length.

## Conclusion

Double arthrodesis is an equally reliable surgical option for PTTI stage III for achieving union, improving the functional outcomes, and deformity correction as triple arthrodesis with a significantly shorter operative time and lower risk of wound complications in the former. The authors recommend double arthrodesis if the calcaneocuboid joint is unaffected and in severe valgus deformities where there is a concern about lateral soft tissues. Long-term outcomes studies with large numbers of patients are required.

## Data Availability

Not applicable.
